# Metagenomics reveals unique gut mycobiome biomarkers in major depressive disorder - a non-invasive method

**DOI:** 10.3389/fcimb.2025.1582522

**Published:** 2025-06-04

**Authors:** Xuan Wang, Di Cao, Wei Chen, Jiaxin Sun, Huimin Hu

**Affiliations:** ^1^ Department of Dermatology, Lianyungang Municipal Oriental Hospital, Lianyungang, China; ^2^ Department of Dermatology, The First People’s Hospital of Lianyungang, Lianyungang, China; ^3^ Department of Dermatology, The Affiliated Huai’an Hospital of Xuzhou Medical University and The Second People’s Hospital of Huai’an, Huaian, China

**Keywords:** major depressive disorder, metagenome, gut mycobiome, machine learning, biomarkers

## Abstract

**Background:**

An increasing amount of evidence suggests a potential link between alterations in the intestinal microbiota and the onset of various psychiatric disorders, including depression. Nevertheless, the precise nature of the link between depression and the intestinal microbiota remains largely unknown. A significant proportion of previous research has concentrated on the study of gut bacterial communities, with relatively little attention paid to the link between gut mycobiome and depression.

**Methods:**

In this research, we analyzed the composition and differences of intestinal fungal communities between major depressive disorder (MDD) and healthy controls. Subsequently, we constructed a machine learning model using support vector machine-recursive feature elimination to search for potential fungal markers for MDD.

**Results:**

Our findings indicated that the composition and beta diversity of intestinal fungal communities were significantly changed in MDD compared to the healthy controls. A total of 22 specific fungal community markers were screened out by machine learning, and the predictive model had promising performance in the prediction of MDD (area under the curve, AUC = 1.000). Additionally, the intestinal fungal communities demonstrated satisfactory performance in the validation cohort, with an AUC of 0.884 (95% CI: 0.7871-0.9476) in the Russian validation cohort, which consisted of 36 patients with MDD and 36 healthy individuals. The AUC for the Wuhan validation cohort was 0.838 (95% CI: 0.7403-0.9102), which included 40 patients with MDD and 42 healthy individuals.

**Conclusion:**

To summarize, our research revealed the characterization of intestinal fungal communities in MDD and developed a prediction model based on specific intestinal fungal communities. Although MDD has well-established diagnostic criteria, the strategy based on the model of gut fungal communities may offer predictive biomarkers for MDD.

## Introduction

Major depressive disorder (MDD) is a multifactorial mental disorder affecting over 35 million people globally, characterized by significant and persistent low mood (Cacheda et al., 2019; Oh et al., 2019). The prevalence of MDD has surged in recent years, particularly due to the SARS-CoV-2 pandemic, which led to a 27.6% increase in 2020s (Liu L. et al., 2023).

Advances in metagenomics have enhanced our comprehension of the intestinal microbiome in human health, particularly regarding its connection to psychiatric disorders. The gut-brain axis, which links the gut microbiota to brain function, has been extensively studied, revealing the role of the microbiome in MDD pathogenesis (Maes et al., 2008; Maes et al., 2012; Maes et al., 2013). Animal studies have shown that gut microbiome alterations can induce depressive-like behaviors, which can be mitigated by probiotics (Clarke et al., 2013; Guida et al., 2018). In humans, fecal microbiota transplantation from MDD patients to rodents induces depressive-like phenotypes, highlighting the role of microbiome in psychiatric disorders (Cheung et al., 2019).

While most research has focused on bacterial dysbiosis in MDD, fungi, although less abundant (< 0.1% of gut microorganisms), play a significant role in gut homeostasis and the host immune system (Qin et al., 2010; Arumugam et al., 2011; Underhill and Iliev, 2014). Recent studies have found associations between intestinal fungi and neurological disorders, such as elevated levels of Candida albicans in MDD patients (Underhill and Iliev, 2014; Zhang et al., 2021; Begum et al., 2022; McGuinness et al., 2024). Fungal dysbiosis may thus serve as a non-invasive biomarker for MDD. As far as we know, no studies have yet utilized gut fungal-associated features as non-invasive biomarkers for depression.

Machine learning, which falls under the category of artificial intelligence, develops predictive models through data analysis. It has been successfully applied to diagnose and predict various diseases, including preterm labor, colorectal cancer, and alcoholic hepatitis (Wang et al., 2024). In this research, we analyzed the intestinal fungal communities of MDD and healthy individuals, constructing a predictive model using machine learning algorithms. We validated this model across diverse cohorts to assess its potential as a non-invasive biomarker, accounting for factors like gender, age, body mass index, medication, and geography (Liu Q. et al., 2023).

## Methods

### Data collection

To avoid introducing bias stemming from the use of various data processing methods, we chose to employ sequence read archives (SRA) instead of processing data outcomes from existing research platforms. In this study, raw metagenomic sequencing data from NCBI (SRA accession numbers: PRJNA1083304, PRJNA762199, and PRJNA943232) were utilized. The PRJNA1083304 training dataset included 20 healthy controls and 16 individuals with MDD. The validation dataset (PRJNA762199) comprised a total of 36 healthy controls and 36 individuals with MDD. The validation dataset (PRJNA943232) included 42 healthy controls and 40 individuals with MDD. All patients in the study satisfied the diagnostic criteria for MDD and these patients were first-onset MDD who had not received treatment. All patients were free of other psychiatric disorders, any somatic illness, and a history of substance abuse. All patients had no history of antibiotic, probiotic, prebiotic or synbiotic administration in the week or three months prior to enrollment.

### Data processing

First, the raw data file is converted from SRA format to FASTQ format using SRA Toolkit’s fastq-dump. The sequencing reads quality was evaluated using the FASTQC tool (Specific parameters: time fastqc seq/*.gz -t 64). Sequencing adapters, low-quality reads, and human DNA contamination were removed using the Kneaddata and Trimmomatic software (Specific parameters: –trimmomatic-options “SLIDINGWINDOW:4:20 MINLEN:50” –bowtie2-options “–very-sensitive –dovetail”). Subsequently, clean sequences were annotated using Kraken2 using the fungal database as the reference. Finally, Bracken was used to estimate the abundance of the gut fungal community.

### Data visualization

The data were normalized using the rarefy_even_depth() function in the R Phyloseq library, and OTUs with relative abundance less than 0.01% were removed. Alpha diversity was performed using the vegan package, with P < 0.05 considered statistically significant, and alpha diversity was visualized using the ggplot2 and ggpubr packages. The beta diversity was performed on the basis of the Bray-Curtis distance, visualized by the Principal Coordinate Analysis (PCoA). Statistical Analysis of Metagenomic Profiles (STAMP) was used to analyze the differences in gut fungal community between MDD and healthy controls. Finally, the support vector machine-recursive feature elimination (SVM-RFE) model was used to search for potential microbial markers that can distinguish between MDD and healthy individuals. The SVM-RFE was then trained on the training set with 10-fold cross-validation.

## Results

### Clinical characteristics in MDD and healthy controls

We included multiple metagenomic sequencing data from different regions as well as countries with the aim of more accurately characterizing the intestinal fungal community of MDD patients. In the training cohort, there were no statistically significant differences between MDD and HCs in sex and body mass index (BMI) (P > 0.05). There was a statistically significant difference in age between MDD and HCs, but the difference in mean age between the two groups was only 3.31 years. We used the Hamilton Rating Scale for Depression (HAMD-17) to assess the level of depression in patients, and the results were statistically significant (P < 0.001). In the validation cohort, MDD and HCs were similar in sex, age, and BMI (P > 0.05) (**Table 1**).

**Table 1:** Clinical characteristics in MDD and HCs.

Group	Sample size	Sex(Male/Female)	Age(years)	BMI	HAMD-17
Training cohort(Shanxi)	MDD (n = 16)	8/8	20.19±4.230	21.68±3.626	26.31±6.620
	HCs (n = 20)	7/13	23.50±3.348	22.17±2.115	1.85±2.720
	t/χ2	–	2.625	0.507	15.054
	P	0.500^a^	0.013^b^	0.615^b^	0.000^b^
Validation cohort(Russia)	MDD (n = 36)	19/17	30.83±10.769	22.39±4.680	21.33±3.610
	HCs (n = 36)	19/17	33.97±10.476	24.63±4.765	1.64±1.944
	t/χ2	0	1.254	1.866	28.822
	P	1.000^a^	0.214^b^	0.067^b^	0.000^b^
Validation cohort(Wuhan)	MDD (n = 40)	5/35	21.15±2.723	20.02±2.114	–
	HCs (n = 42)	8/34	22.00±2.828	20.71±2.253	–
	t/χ2	0.658	1.385	1.42	–
	P	0.417^a^	0.170^b^	0.160^b^	–

a P value for chi-square test.

b P values for two-sample t-test.

### Comparison of the intestinal fungal community diversity in MDD versus healthy controls

**Figure 1:**
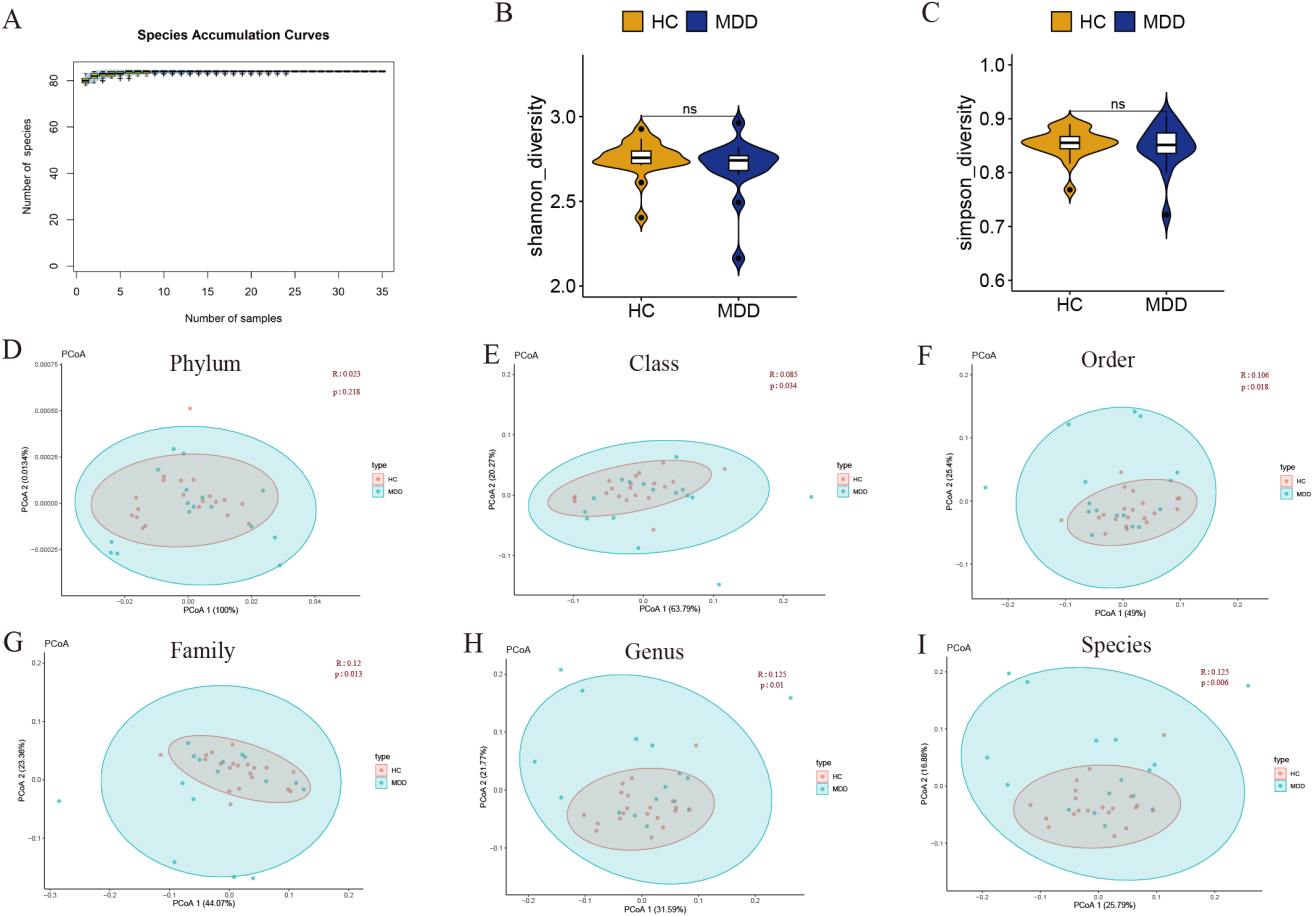
**(A)** Species accumulation curve of the intestinal fungal communities. **(B, C)** Alpha diversity reflected the richness and evenness between MDD and healthy controls. Statistical analysis was performed using the Kruskal-Wallis test and Wilcoxon test. Ns represents not statistically significant. Yellow represents healthy controls (n=20) and blue represents the MDD (n=16). **(D-I)** Beta-diversity analysis of the intestinal fungal communities between MDD and healthy controls by using principal coordinate analysis (PCoA) based on Bray-Curtis. The significance of clustering was determined using analysis of similarities (ANOSIM). Red represents healthy controls (n=20) and blue represents the MDD (n=16). P < 0.05 was considered statistically significant.

After analysis of the metagenomic sequencing data, a total of 1,648,320 reads associated with the fungus were obtained from 36 libraries. The species accumulation curve analysis demonstrated that the curves reached a plateau, indicating that the sample size was sufficient to elucidate the characteristics of the fungal microbiome (**Figure 1A**).

We used the Shannon-Wiener index and Simpson index to assess the fungal alpha diversity between MDD and healthy controls. There was no statistically significant difference in the Shannon-Wiener index and Simpson index between the two groups (P > 0.05) (**Figures 1B, C**). This suggested that the species richness and uniformity are consistent in MDD and healthy controls. There may be selection bias due to the small sample size in the study, and the results still need to be further verified by large samples.

Subsequently, beta diversity was calculated based on the Bray-Curtis distance and was measured using principal coordinate analysis (PCoA). The unweighted UniFrac analyses indicated that the PCoA could distinguish between the healthy controls and MDD groups at the class, order, family, genus, and species levels (**Figures 1E-I**). However, no significant differences were observed between the two groups at the phylum level (**Figure 1D**). The results of beta diversity indicated that the gut fungal communities of individuals with MDD and healthy controls are distinct.

### Screening for fungal biomarkers to differentiate MDD from healthy controls

**Figure 2:**
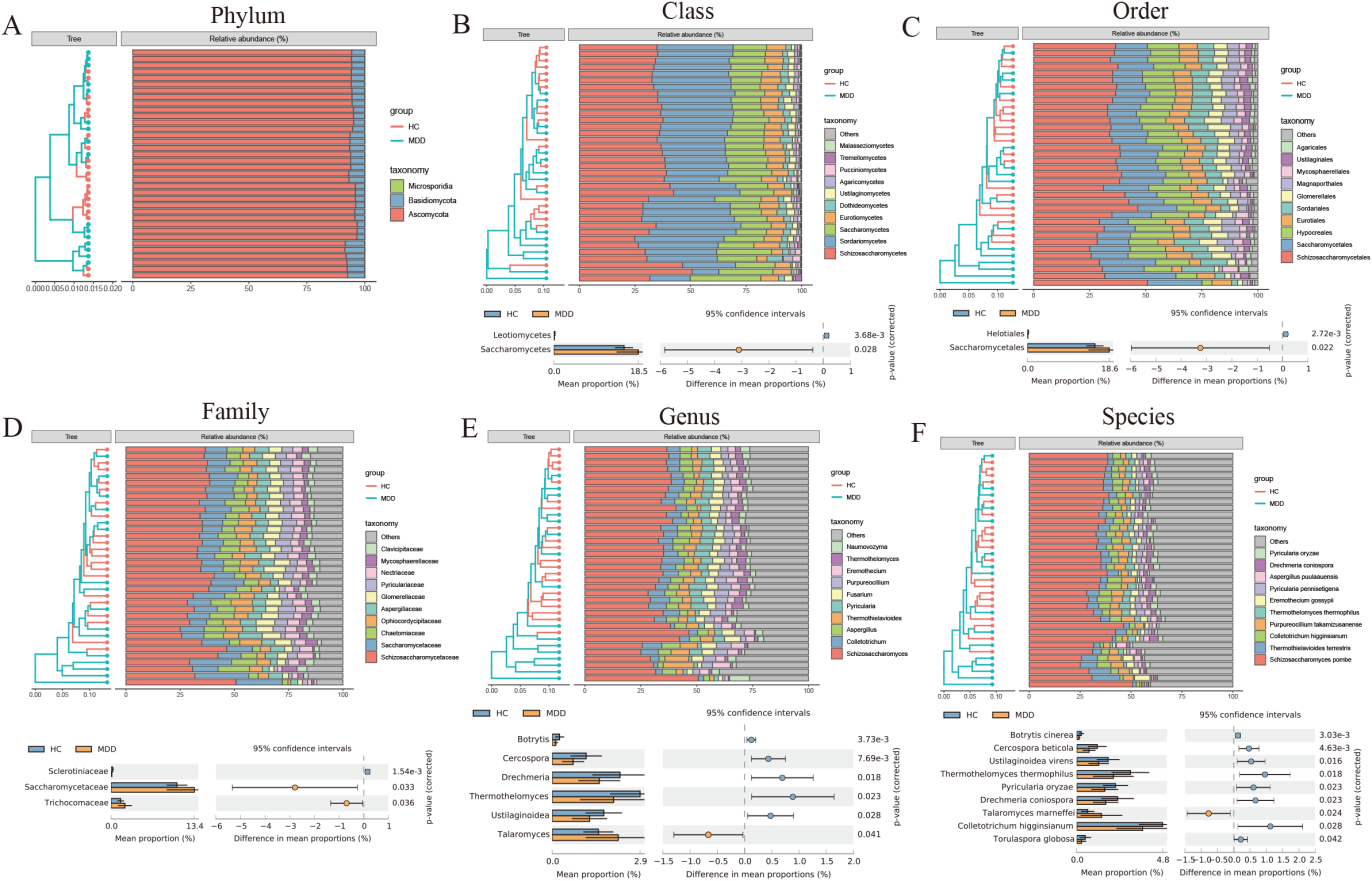
**(A-F)** The composition and differences of the intestinal fungal communities between MDD and healthy controls using STAMP (two-sided Welch t-tests).

A total of 22 differential fungal communities were identified using Statistical Analysis of Metagenomic Profiles (STAMP). Six fungal communities were found to be enriched in MDD, including Saccharomycetes, Saccharomycetales, Saccharomycetaceae, Trichocomaceae, Talaromyces, and Talaromyces marneffei. Conversely, 16 fungal communities were significantly and markedly reduced, including Leotiomycetes, Helotiales, Sclerotiniaceae, Botrytis, Cercospora, Drechmeria, Thermothelomyces, Ustilaginoidea, Botrytis cinerea, Cercospora beticola, and others (**Figures 2A-F**).

**Figure 3:**
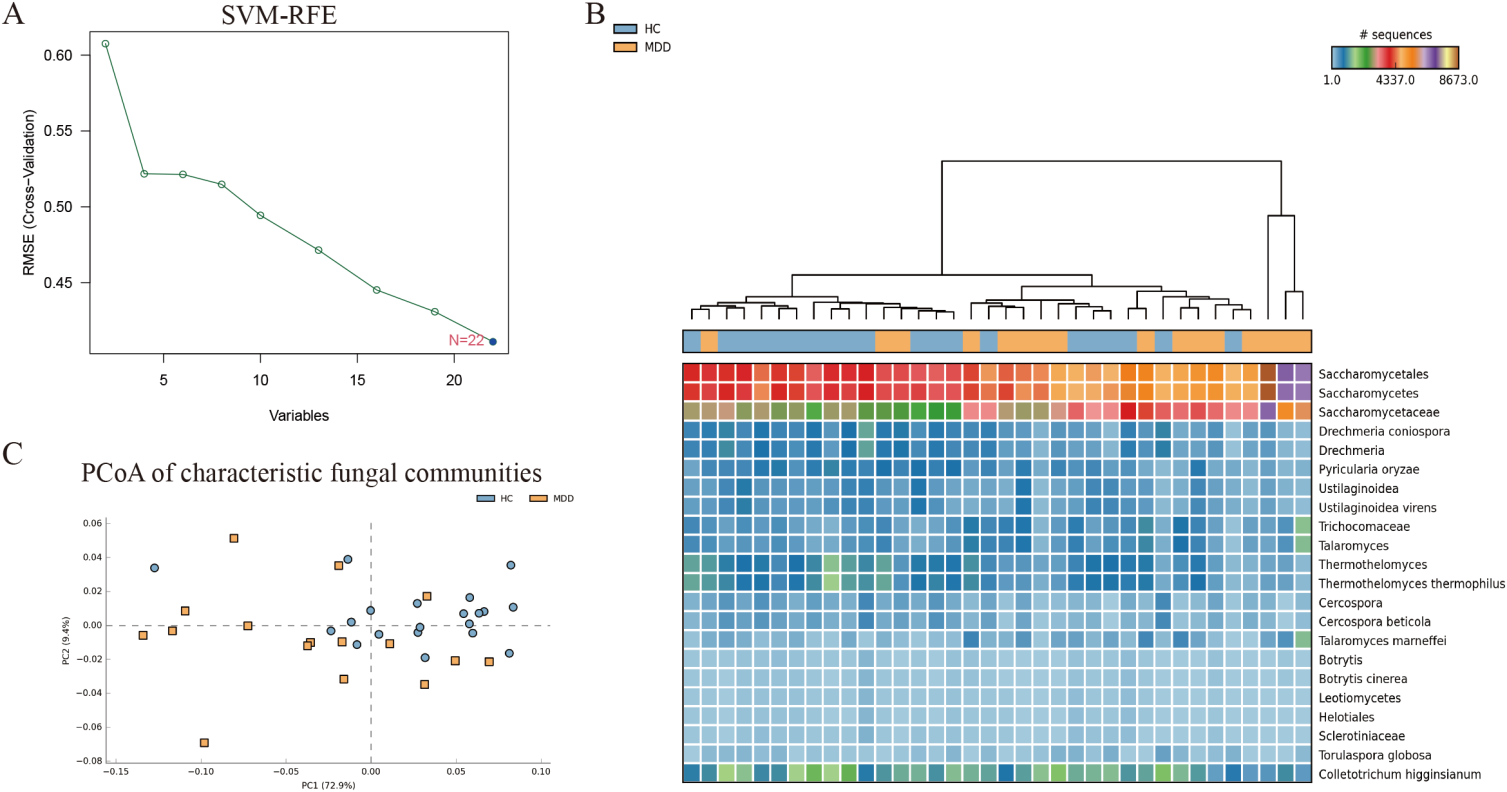
**(A)** SVM-REF algorithm screening for characteristic fungal communities. **(B)** Heat map of the 22 characteristic fungal communities. **(C)** PCoA of the 22 characteristic fungal communities.

The support vector machine-recursive feature elimination (SVM-RFE) algorithm was used to identify fungal biomarkers. The SVM-RFE algorithm demonstrated optimal classification accuracy when the number of features was set to 22. Ultimately, 22 distinctive fungal communities were identified (**Figure 3A**). In the heatmap, we can observe the difference in the abundance of the 22 fungal communities between MDD and the healthy groups (**Figure 3B**). Meanwhile, the PCoA results showed that characteristic fungal communities could distinguish healthy individuals with MDD (**Figure 3C**).

**Figure 4:**
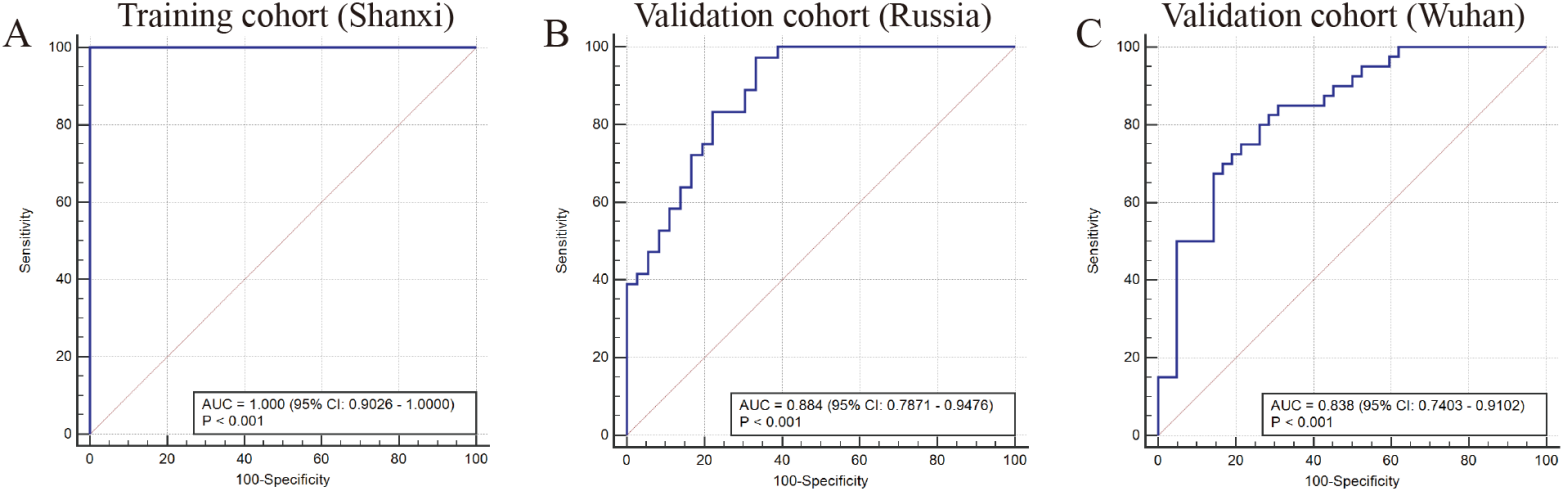
**(A)** ROC curve (AUC) in training cohort from Shanxi (HCs n=20; MDD n=16). **(B)** ROC curve in the validation cohort from Russia (HCs n=36; MDD n=36). **(C)** ROC curve in validation cohort from Wuhan (HCs n=42; MDD n=40).

Finally, we assessed the predictive efficacy of fungal communities for MDD via receiver operating characteristic (ROC) curves. In the training cohort, the area under the receiver operating characteristic curve (AUC) was 1.000 (95% CI: 0.9026-1.0000). This finding suggests that these characteristic fungal communities can serve as a valuable reference indicator for MDD (**Figure 4A**). To further substantiate the predictive efficacy of fungal communities, external validation was performed using two independent cohorts from Russia and Wuhan, with the objective of confirming the reliability of the model. The AUC of the Russian validation cohort was 0.884 (95% CI: 0.7871-0.9476) with good predictive ability (**Figure 4B**). The AUC for the Wuhan validation cohort was 0.838 (95% CI: 0.7403-0.9102) with good predictive ability (**Figure 4C**).

## Discussion

In this research, we analyzed the intestinal fungal communities in MDD and identified 22 differential fungal communities at various taxonomic levels using STAMP. We developed an SVM-RFE machine learning model to screen potential fungal markers related to MDD, offering a novel approach to predict depression (Hu et al., 2019).

The gut-brain axis, which forms a bidirectional regulatory system between the brain and the gut through neuroendocrine, neuroimmune, and neuroanatomical pathways, has received increasing attention in neurodevelopmental, psychiatric, and neurodegenerative disorders (Zhang et al., 2018; Hu et al., 2019). Gut flora is capable of producing neurotransmitters, short-chain fatty acids, branched-chain amino acids, and gut hormones, which can influence brain function and behavior. The ability of gut flora to regulate tryptophan metabolism and synthesize neurotransmitters like dopamine, norepinephrine, gamma-aminobutyric acid, and acetylcholine underscores its role in mental health (Wu et al., 2020; Prochazkova et al., 2021). Chemicals released from gut microorganisms can directly alter the electrical activity of the vagus nerve and vagus-innervated brain regions, potentially contributing to depression (Plaza-Díaz et al., 2019; Namgung et al., 2022; Rea et al., 2022).

Although the association between depression and gut bacteria is well-documented, the role of fungi remains to be further explored (Song et al., 2019; Tian et al., 2022). The fungal microbiome, though a minor component of the intestinal microbiota (Qin et al., 2010; Arumugam et al., 2011), plays a pivotal role in host health and microbe-microbe interactions (Nash et al., 2017). Clinical studies have revealed significant changes in gut fungal communities in individuals with neuropsychiatric disorders, including depression (Hao et al., 2023).

The study of the gut mycobiome has been limited due to technical and analytical challenges (Jiang et al., 2020). However, advancements in bioinformatics have facilitated the identification of fungi, expanding our understanding of their role in human health and disease (Taylor and Houston, 2011; Jha et al., 2021). Our metagenomic analysis of 16 MDD patients and 20 healthy individuals revealed significant differences in gut fungal community composition, despite no significant difference in alpha diversity. The result may be influenced by a number of factors, including differences in strain abundance, disease assessment, and flora dynamics (Richard et al., 2015; Wang et al., 2015; Vižlin et al., 2024). In a systematic review of clinical trials and observational studies, researchers compiled data from 35 studies, and about 2/3 of the studies showed no significant difference in alpha diversity between MDD and HCs. Recent studies of the human gut microbiome suggest that alpha diversity metrics have limited utility in distinguishing between healthy and diseased populations. The results of several studies have shown significant differences in beta diversity between MDD and HCs. These studies are consistent with the results of our study (Larsen and Claassen, 2018; Alli et al., 2022).

Given the dynamic nature of the gut fungal community, a single fungal taxon cannot serve as a reliable biomarker for disease diagnosis (Strati et al., 2017). Therefore, we trained machine learning (ML) algorithms based on differential gut fungal communities to improve disease prediction (Wang et al., 2024). Recent similar studies have effectively utilized SVM-RFE to identify COMMD9, CSF3R, and NUB1 as potential biomarker genes for predicting sepsis, uncovering new mechanisms in disease pathogenesis that may offer opportunities for therapeutic intervention (Wang et al., 2023). Similarly, SVM-RFE was employed to identify 12 Helicobacter pylori (HP) hub genes closely associated with gastric cancer, which may aid in the molecular diagnosis and personalized treatment of gastric cancer (Luo et al., 2023). We constructed an SVM-RFE model, which demonstrated excellent predictive performance in discriminating between MDD patients and healthy individuals. A total of 22 fungal biomarkers were identified, of which 6 were significantly increased in MDD. These 6 fungal species play important roles in immune regulatory and anti-inflammatory effects (**Table 2**) (Nagpal et al., 2020; Bukavina et al., 2022; Lei et al., 2022; Shuai et al., 2022; Zhang H. et al., 2022; Zhang et al., 2023). The metabolic activity of fungal communities may be associated with the development of major depressive disorder. Further studies of gut fungal communities are needed to determine how fungi affect host health.

**Table 2:** Features or functions of characteristic fungal communities in MDD.

Species	Features or Functions
Saccharomycetes	A major component of the human gut microbiota and exhibit immune regulatory and anti-inflammatory effects by inducing interleukin-10 production.
Saccharomycetales	Saccharomycetales were significantly positively correlated with short-chain fatty acids (SCFA) production.
Saccharomycetaceae	Fungi such as Saccharomycetaceae play an important role in autoimmune responses through interactions with bacteria.
Trichocomaceae	The abundance of Trichocomaceae is significantly increased in the gut of patients with psychiatric disorders such as mild cognitive impairment.
Talaromyces	Studies have shown that Talaromyces produce kinds of secondary metabolites, some of which have biological activities such as anti-inflammatory, bacteriostatic, and antitumor activities.
Talaromyces marneffei	Talaromyces marneffei is the only pathogenic fungus in its genus that inhibits the host pro-inflammatory response through Mp1p binding to arachidonic acid, thereby evading the host immune response.

Cross-regional validation, considering dietary and geographic factors, further demonstrated the model’s validity and applicability. Consistent with gut bacteria, gut fungi exhibit dynamic changes throughout an individual’s life, influenced significantly by geography, diet, and host factors such as gender, age, and drug use (Yan et al., 2024). A large-scale population-based survey across China, encompassing six ethnic groups, underscored the substantial impact of geography and ethnicity on gut fungal composition (Zhang F. et al., 2022). In our study, validation cohorts from diverse regions demonstrated robust results, highlighting the potential of gut fungi as microbial markers for depression and the broad applicability of our methodology across different geographic areas.

## Conclusion

In this research, we elucidated the characterization of the gut fungal communities of patients with MDD, used the SVM-RFE algorithmic model to screen for fungal markers associated with MDD, and validated the prediction effect in a cohort from different regions. Notably, despite the possibility of misdiagnosis, our study demonstrates the potential of using gut fungal communities to train supervised SVM-RFE models for depression diagnosis. We hope that this study will better assist clinicians in diagnosing depression for the further benefit of patients. The limitation of this study is the lack of data on metabolites from the mycobiome. Due to the limited sample size, further research is necessary to ascertain the generalizability of the study’s findings.

## Data Availability

The datasets presented in this study can be found in online repositories. The names of the repository/repositories and accession number(s) can be found in the article/supplementary material.
